# Salt-Bridge Energetics in Halophilic Proteins

**DOI:** 10.1371/journal.pone.0093862

**Published:** 2014-04-17

**Authors:** Arnab Nayek, Parth Sarthi Sen Gupta, Shyamashree Banerjee, Buddhadev Mondal, Amal K. Bandyopadhyay

**Affiliations:** 1 The Department of Biotechnology, The University of Burdwan, Burdwan, West Bengal, India; 2 Department of Zoology, Burdwan Raj College, The University of Burdwan, Burdwan, West Bengal, India; Russian Academy of Sciences, Institute for Biological Instrumentation, Russian Federation

## Abstract

Halophilic proteins have greater abundance of acidic over basic and very low bulky hydrophobic residues. Classical electrostatic stabilization was suggested as the key determinant for halophilic adaptation of protein. However, contribution of specific electrostatic interactions (i.e. salt-bridges) to overall stability of halophilic proteins is yet to be understood. To understand this, we use Adaptive-Poison-Boltzmann-Solver Methods along with our home-built automation to workout net as well as associated component energy terms such as desolvation energy, bridge energy and background energy for 275 salt-bridges from 20 extremely halophilic proteins. We then perform extensive statistical analysis on general and energetic attributes on these salt-bridges. On average, 8 salt-bridges per 150 residues protein were observed which is almost twice than earlier report. Overall contributions of salt-bridges are −3.0 kcal mol^−1^. Majority (78%) of salt-bridges in our dataset are stable and conserved in nature. Although, average contributions of component energy terms are equal, their individual details vary greatly from one another indicating their sensitivity to local micro-environment. Notably, 35% of salt-bridges in our database are buried and stable. Greater desolvation penalty of these buried salt-bridges are counteracted by stable network salt-bridges apart from favorable equal contributions of bridge and background terms. Recruitment of extensive network salt-bridges (46%) with a net contribution of −5.0 kcal mol^−1^ per salt-bridge, seems to be a halophilic design wherein favorable average contribution of background term (−10 kcal mol^−1^) exceeds than that of bridge term (−7 kcal mol^−1^). Interiors of proteins from halophiles are seen to possess relatively higher abundance of charge and polar side chains than that of mesophiles which seems to be satisfied by cooperative network salt-bridges. Overall, our theoretical analyses provide insight into halophilic signature in its specific electrostatic interactions which we hope would help in protein engineering and bioinformatics studies.

## Introduction

The family halobactereaceae or halophiles are archaea that thrive in natural habitat of saturated brine [1] and pH optima in neutral range. Intracellular salt concentration is similar to that in the environment outside the cell. Thus, the entire protein machinery of halophiles is dependent on high salt concentration for function and stability [2], [3]. In general, high concentration of salt is detrimental to mesophilic proteins. It enhances aggregation and collapse of 3D structure of proteins. It also interferes with electrostatic interactions due to charge screening and reduces natural hydration of proteins [1]. In contrast to its mesophilic counterpart, halophilic proteins maintain structural and functional integrity only in saturated salt solution, withdrawal of which causes gradual loss of tertiary structure. At low salt condition such unfolding is caused both by non-specific electrostatic and hydrophobic destabilization [1], [4], [5].

How then halophilic proteins remain stable in high salt environment? Genome, proteome-wide analyses as well as studies on specific halophilic proteins showed a number of compositional biases in their sequences. In general, higher abundance of acidic over basic residues, low content of bulky hydrophobic residues over less bulky ones in sequences of halophilic proteins are observed [1], [6]–[10]. In 3D structures, majority of these acidic residues are found specifically positioning on the surface of proteins which were proposed to facilitate excess protein hydration thereby making the surface less hydrophobic, more flexible and thus help to overcome deleterious effect of salt by promoting non-specific electrostatic interactions with salts in solution [10], [11]. Detailed studies on malate dehydrogenase from *Haloarcula marismortui* confirmed interactions among surface acidic residues with hydrated salt ions which were argued to prevent aggregation [12] and also help to achieve functional state of protein. Similarly clusters of acidic residues are observed on the surface of atomic structure of dihydrofolate reductase, proliferating cell nuclear antigen (PCNA) from *Haloferax volcanii* [13], [14] and glucose dehydrogenase from *H. mediterranei* [15]. Further, reduction of hydrophobic surface is achieved by another novel strategy by making surface of these proteins deficit of lysine residue [7], [9], [15].

Physical Chemistry and native state of halophilic proteins are made by conventional weak interactions of which non-specific classical electrostatic interactions have received the major focus [1], [13], [16], [17]. Salt and pH dependent spectroscopic studies on halophilic ferredoxin from *Halobacterium salinarum* showed high salt not only contribute to classical electrostatic stability but also play role in solvent mediated stabilization [18]. It has been argued that classical electrostatic stability is saturated at a salt concentration of ∼0.1M and thus high salt (4M) is needed for maintaining hydrophobic interactions [1], [18]. Crystal structure analyses of Malate dehydrogenase from *Haloarcula marismortui* [19] showed greater number of salt-bridges than its mesophilic counterpart which enhanced enzyme stability at high salt concentrations. In pH dependent urea induced kinetic studies of the native state of ferredoxin from *Halobacterium salinarum* showed optimal kinetic stability at neutral pH which becomes unstable at either acidic or alkaline pH indicated presence of stabilizing salt-bridge in this protein [20].

Computational studies based on PBE, a pioneer theoretical analysis was carried out on crystal structure of ferredoxin and malate dehydrogenase from *Haloarcula maismortu* for understanding contribution of salt and pH on classical electrostatic stability. Further, in comparative analyses of halophilic and non-halophilic proteins, it has been demonstrated that the former gain stability with increment of salt concentration or decrease of pH in low salt [21].

Ion-pair or salt-bridge is one major contributor for the stability of proteins in general [22], [23]. It is more so in case of proteins adapted in extreme of environment such as at high salt or temperature [19], [20], [24], [25]. Either experimental or theoretical determination of interaction energy of salt-bridges shows that they could either be stabilizing [26]–[29] or destabilizing [30]–[32]. Energy of salt-bridge can be partitioned into three component terms such as columbic attraction of opposite charges, their desolvation and background interactions. The first term is always contributing and other two terms could either be contributing or costly. The favorable charge-charge attraction within a salt-bridge is often opposed by the unfavorable desolvation of charges and is further modulated by charge-dipole interactions as well as by the ionization behavior of nearby charge groups [31]. Thus the net energy of a given salt-bridge could either be stabilizing [26]–[29] or destabilizing [30]–[32] or insignificant [33] and the same are entertained both in experimental or theoretical scenario. As far as calculation of net energy of salt-bridge is concerned, computational procedure is advantageous over experimental Pka and double mutation cycle methods, in that separation of direct and indirect terms as well as pH and ionic strength variation are possible for a given set of parameters and structural model of proteins [29], [31], [34]. Poisson-Boltzmann Equation (PBE) is an ideal continuum electrostatic descriptor for bimolecular system and thus its solvers methods such as Delphi [24], [25], [28], [29], [31], [35] and APBS [36] are most popularly used.

Here we present results of systematic and extensive analysis of involvement of acidic and basic residues in the formation of monomeric and network salt-bridges and their contribution to overall stability in the native state of proteins from a dataset of 20 high resolution (≤1.4 Å) crystal structures from halophilic domain possessing a total of 275 non-equivalent salt-bridges. Frequencies of these salt-bridges for each of six pairing partners (such as Arg-Asp, Arg-Glu, Lys-Asp, Lys-Glu, His-Asp and His-Glu) and their presence in secondary structures are also been worked out. We also report computation of different component energy terms using APBS methods for: (a) determination of net contribution of salt-bridges in halostability, (b) binary classification of sat-bridges into stable or unstable exposed or buried, isolated or networked and H-bonded or non-H-bonded categories and (c) establishing correlation of average accessibility of salt-bridges with these energy terms. Our present study shows details of salt-bridge energetic of halophilic proteins, knowledge of which has potential implication in comparative bioinformatics and protein engineering.

## Results and Discussion

### General characteristics of salt-bridges and its partners in halophilic proteins

Halophilic proteins are reported to possess excess of negative charges over basic residues that contribute to the overall stability by non-specific electrostatic interactions [1], [3], [7], [9], [10], [12], [14], [21]. However, such electrostatic interactions, which are saturated at around 0.1M NaCl, was reported to make less contribution to the overall stability of halophilic proteins [1]. Again lower content of bulky hydrophobic residues [1], [9] that are also present under low water activity situation in saturated brine solution [37], hydrophobic force seems to have lower contribution to halostability. Thus, arguably specific electrostatic interactions which are less affected by the presence of multimolar salts [22] seem to have major contribution to the stability of halophilic proteins. To the best of our knowledge, details of involvement and contributions of salt-bridges and their energetics using computational approach involving crystallographic structures are absent. We therefore used 20 high resolution halophilic protein structures to understand salt-bridge energetics using PBE solver methods [38].

Table 1 show acidic and basic residues extracted from 20 unique chains (i.e. A chain) of 20 crystal structures and their participation in salt-bridge formation. Following points are noteworthy from the table. Firstly, although normalized composition of acidic residue (19.76%) exceeds than that of basic residues (11.09%), lower fraction of the former (27.58%) than the later (40.3%) participates in salt-bridge formation. It is worth noting here that acidic residues participate both in non-specific electrostatic as well as salt-bridge stability. However, these unequal normalized frequencies of acidic and basic residues participating in salt-bridge formation also indicate presence of network salt-bridges. Secondly, among basic residues, Arg has higher abundance and also contribute greater fraction of it for salt-bridge formation. In turn Glu which has lower abundance than Asp, contributes higher fraction in salt-bridge formation. The preferences of basic and acidic residues for salt-bridge formation as seen above might have relation with their side chain structure, length, relation to the stability of secondary structures and accessibility.

**Table 1:** Absolute and normalized frequency of total and salt-bridge forming acidic and basic residues as obtained from 20 halophilic proteins (see Materials and Methods).

Residues/class	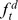	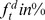	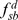	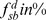	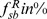
Arg	279	5.61	127	2.55	45.45
Lys	144	2.89	57	1.14	39.44
His	129	2.59	39	0.78	30.11
Basic	552	11.09	223	4.47	**40.30**
Asp	501	10.07	126	2.53	25.12
Glu	482	9.69	145	2.92	30.13
Acidic	983	19.76	271	5.45	**27.58**
Total	1535	30.85	494	9.92	32.16

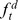
: Residues absolute frequency in 20 protein chains in the database. 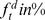: Percent frequency w.r.t. total residues (4975). 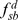: Residues salt-bridge absolute frequency. 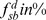: Percent salt-bridge frequency w.r.t total residues. 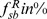: Percent salt-bridge frequency w.r.t. corresponding salt-bridge forming residues. Total residues in 20 unique chains 4975.

**Figure 1:**
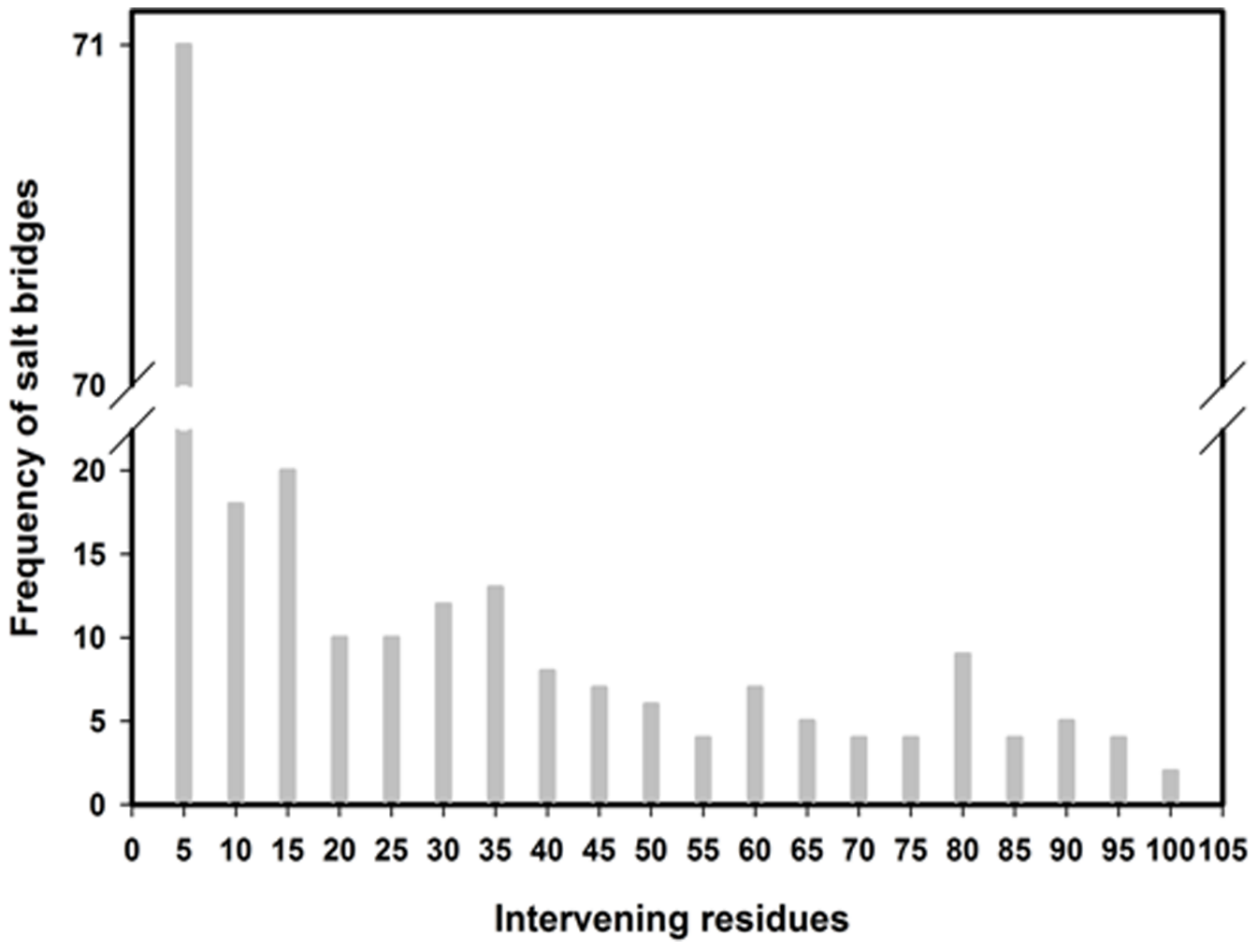
Histogram showing the frequency of salt-bridges against intervening residues for first 100 residues from N-terminal region of 20 halophilic proteins. This length is common for all proteins in our database.

To check salt bridging partner's positional distribution in protein sequences, we have made grouped frequency distribution plot of intervening residue distances for a class interval of 5 and the same is plotted in figure 1. In this plot we have consider only first 100 residues for each of 20 proteins. Thus, the grouped frequency for each class interval represents their value for the overlapping region of 20 protein sequences. The plot shows that salt-bridging acidic and basic residues when present closer in sequence tend to form more number of salt-bridges. The frequency decreases with increase in intervening residues between the bridging partners. Our observation in context of halophilic proteins obtains the similar pattern as was entertained earlier in the context of mesophilic proteins [29]. This kind of coding pattern in relation to specific electrostatic interactions which is seen in halophilic (present study) and mesophilic cases [29] are reminiscent of hierarchal protein folding [39], [40].

**Figure 2:**
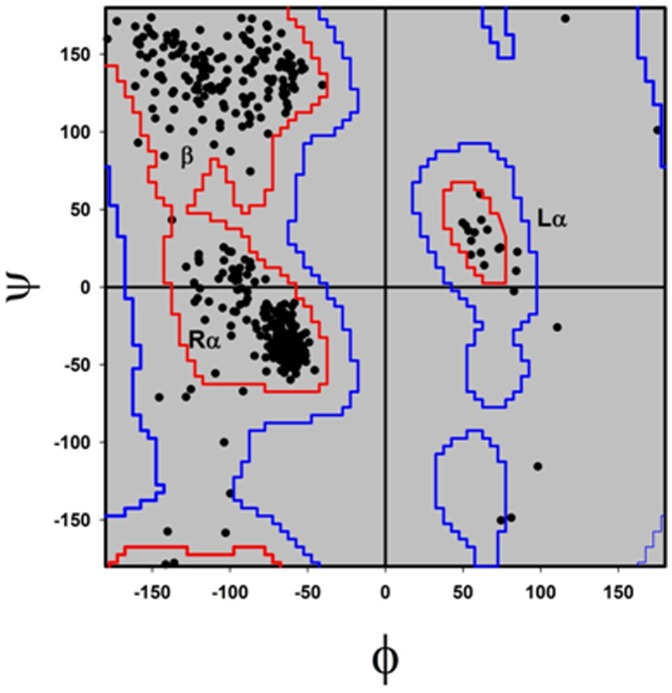
Ramachandran plot of salt-bridge forming residues (275×2). In this plot core region is outlined in blue and allowed region in red. These boundary values are taken from Lovell et. al., 2003 [52].

In general, protein structure is more conserved than their primary sequences. Secondary structure [41] which determines protein topology is thus more conserved in evolution. Earlier studies with salt-bridges showed that major fraction of these residues are distributed in secondary structure region [29]. Are salt-bridges conserved in halophiles? The question is justified as halophilic proteins to adapt in extreme of salt solution, have to pass through critical transition of evolution in relation to salt with reference to its mesophilic counterparts. In this context we were interested to observe the distribution of salt-bridging candidates in secondary structures. Main chain dihedral angles of salt-bridge partners were extracted from energy minimized structures for all 20 halophilic proteins and plotted in figure 2. The plot shows the distribution of dihedral angles (φ,ψ) for salt-bridge partners occupying mostly the defined right handed α-helix (Rα), beeta sheet (β) regions. About 78% of these residues fall in these conserved regions of Ramachandran plot. Such secondary structure specific distribution of salt-bridges not only indicates that they are conserved in halophilic evolution but also highlights their specific nature of interactions.

Table 2 shows frequency of all possible salt-bridge pairs such as Lys-Asp, Lys-Glu, Arg-Asp, Arg-Glu, His-Asp and His-Glu. Arg has highest pairing frequencies with both the acidic residues than Lys and His. Again, in all cases of Asp and Glu, the later is used more in number for formation of salt-bridges by basic residues. As far as distribution of Arg mediated salt-bridges in the core and on the surface is concerned, this residue is favored over Lys. The difference in the proportions of Glu and Asp that are buried in the protein interior is not so large. These facts are in line with earlier observations [40].

**Table 2:** Frequency salt-bridges formed by each of six possible pairs.

Residues	Asp	Glu
Lys	28	38
Arg	82	84
His	18	25

### Observations of all 275 salt-bridges of halophilic proteins in our dataset

Salt-bridge is specific electrostatic interaction that contributes to the overall stability of native state of proteins. The fact that halophilic proteins are devoid of bulky hydrophobic residues [1] and non-specific electrostatic interactions cause marginal stability; specific electrostatic interactions which are less affected by the presence of multi molar salt concentrations [22] are expected to make effective contributions to the stabilization. We use APBS methods along with our in-house automation for calculation of all three associated energy terms (such as **ΔΔG_dslv_**: desolvation, **ΔΔG_brd_**: bridge and **ΔΔG_prt_**: background) for finding net salt-bridge energy (**ΔΔG_tot_** i.e. sum of the above three component terms). **ΔΔG_dslv_** and **ΔΔG_prt_** are indirect interaction terms of which the former is an unfavorable term that originates due to desolvation of charges during folding and the later is due to interaction of charges could either be favorable or unfavorable. **ΔΔG_brd_** is a direct term that always causes favorable contribution due to interactions of charges in the folded state [29].

Heterogeneity in databases might affect statistical generality [42], we therefore involved homogenous dataset of 275 salt-bridges obtained strictly from extremely halophilic proteins. Our observations suggest that halophilic proteins utilize more of specific electrostatic interactions than mesophilic ones (that includes prokaryotic and eukaryotic proteins) studied earlier [29] in that in the former 275 salt-bridges from 20 proteins making on average 13.8 salt-bridges per protein and in the later 222 salt-bridges from 36 proteins making on average 6.2 salt-bridges per protein.

Net salt-bridge energy, on average, is −3.0 (±4.0) kcal mol^−1^ which is contributed almost equally by bridge (**ΔΔG_brd_** = −6.9±4.0 kcal mol^−1^) and background (**ΔΔG_prt_** = −6.7±6.0 kcal mol^−1^) energy terms and unfavored by desolvation (**ΔΔG_dslv_** = 10.6±6 kcal mol^−1^) term (Table 3). The average equal favorable contribution of both bridge and background energy terms is contrasted by earlier observations that, on average, background term (**ΔΔG_prt_** = −3.9±4.0 kcal mol^−1^) contributes only about half the value of bridge term (**ΔΔG_brd_** = −6.3±4.0 kcal mol^−1^) [29]. Overall, desolvation cost is overbalanced by the sum of bridge and background terms.

**Table 3:** Average energy terms in various salt-bridge categories.

		Energy terms in Kcal per Mol.			
Salt-bridge class		ΔΔGdslv	ΔΔGbrd	ΔΔGprt	ΔΔGtot
1	All	+10.57±5.50	−6.88±4.10	−6.66±5.74	−2.96±4.06
2	Stable	+10.91±5.36	−7.54±3.92	−7.72±5.85	−4.35±3.47
	Unstable	+9.41±5.81	−4.60±3.87	−2.99±3.34	+1.82±1.50
3	Buried	+14.87±6.07	−9.14±4.69	−9.85±6.46	−4.13±4.39
	Exposed	+8.34±3.51	−5.70±3.17	−5.00±4.50	−2.35±3.74
4	Isolated	+9.22±5.33	−6.66±4.13	−3.84±4.06	−1.28±2.91
	Networked	+12.20±5.27	−7.14±4.05	−10.3±5.63	−4.97±4.32
5	H-bonded	+11.24±5.32	−7.82±3.83	−6.79±5.66	−3.38±4.00
	No H-bonds	+7.47±5.24	−2.50±1.81	−6.03±6.02	−1.04±3.74

All: whole dataset of 275 salt-bridges; Stable: 213 salt-bridges with ΔΔG_tot_<0 Kcal/mol; Unstable: 62 salt-bridges with ΔΔG_tot_>0 Kcal/mol; Buried: 94 salt-bridges with average ASA of ≤20%; Exposed: 181 salt-bridges with average ASA of >20%; Networked: 125 salt-bridges that participate in salt-bridge networks; Isolated: 150 salt-bridges that do not form part of salt-bridge networks; H-bonded: 226 salt-bridges containing at least one hydrogen bond between side-chain charged groups; and Non H-bonded: 49 salt-bridges that do not contain any hydrogen bond between their side-chain charged groups.

**Figure 3:**
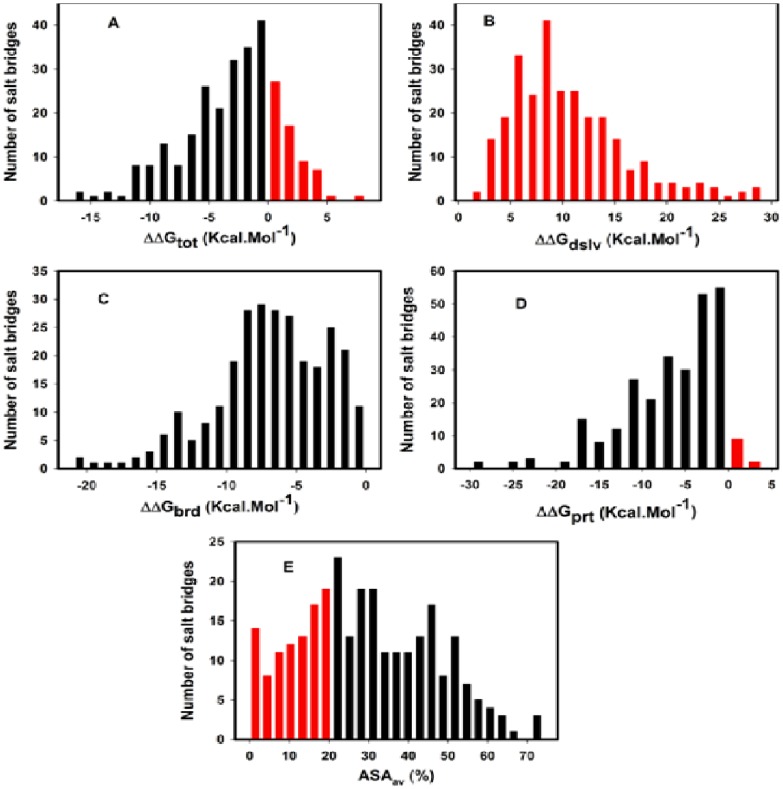
Histogram showing distribution of 275 salt-bridges from 20 halophilic proteins extracted using SBION [53] as a function of ΔΔG_tot_ Kcal Mol^−1^ (A), ΔΔG_dslv_ Kcal Mol^−1^ (B), ΔΔG_brd_ Kcal Mol^−1^ (C), ΔΔG_prt_ Kcal Mol^−1^ (D) and ASA_av_ (%) (E). Figure (A) through (D), black bar indicates stabilizing (i.e. ΔΔG_tot_<0) and red bar indicates destabilizing (i.e. ΔΔG_tot_>0) salt-bridges. Figure (E) red bar (ASA_av_%≤20) indicates salt-bridges present in the core.

To obtain more realistic view on energy contribution of 275 candidate salt brides, distribution of various energy terms are shown in the Figure 3 (A through E). Almost symmetric distribution of **ΔΔG_tot_** term containing both stable and unstable salt-bridges, majority of which are falling in the stabilizing zone (**ΔΔG_tot_**<0) with a maximum near zero from the negative side is observed (figure 3A). Quantitatively 78% (213 of 275) of candidate salt-bridges in our dataset contribute to stability and rest 22% (62 of 275) cause destabilization. Profiles of component energy terms shows that the distribution of bridging (Figure 3C) and desolvation (Figure 3B) terms occupying stabilizing and destabilizing zone respectively while the background term (Figure 3D) possesses both stabilizing (major fraction) and destabilizing population of salt-bridges. This would mean that both bridge and background term act favorably and thus overcome unfavorable desolvation penalty to make net salt-bridge energy stabilizing. The observation that the background term i.e. **ΔΔG_prt_**, which originates due to charge-dipole interactions, contains both stabilizing (major fractions) and destabilizing populations indicate it is most sensitive to protein microenvironment among all component terms. An inspection of these individual terms shows that only 4% (11 of 275) are destabilizing with an average destabilization of 0.7±0.8 kcal mol^−1^ which is much less (11.3% with an average destabilization of 0.9 kcal mol^−1^) than earlier observations [29].

This fact implore us to make closer look on 62 destable salt-bridges in our dataset (see above) for all three component terms for their nature of contribution (favorable or unfavorable) to net stability. In this population both bridge and background terms are still negative, except the above 11 background terms which are positive. However, the magnitude of the negative values are far weaker than stable cases (213 of 275) such that their collaborative effect could not compensate the desolvation cost, indicating both these two terms are responsive to protein environment. In comparison to bridge term, background term seems to be more sensitive to protein microenvironment as some of these values (11 individuals of 62 destable cases) are positive. However our observation (mentioned above), unlike earlier one [29], that overall contributions of these two energy terms to net stability are seen to be almost equal (Table 3). As far as protein environment is concerned, halophilic proteins possess unique design in that in sequences polar as well as negatively charged residues dominate over bulky hydrophobic residues [1] and in structures majority of these residues are distributed on the surface [37]. In halophilic proteins, lower content of bulky hydrophobic residues might indicate mandatory more polar protein interior than their mesophilic counterparts. Thus the context and composition of such excessive charges and dipoles constitute local microenvironment around individual salt-bridge partner which determine the magnitude of their contribution to net stability. In our observations 107 instances of 275 salt-bridges where favorable contribution of background term exceed than that of bridge term for overcoming desolvation penalty and thus making salt-bridges stabilizing: an unique phenomena for halophilic proteins than mesophilic ones [29].

**Figure 4:**
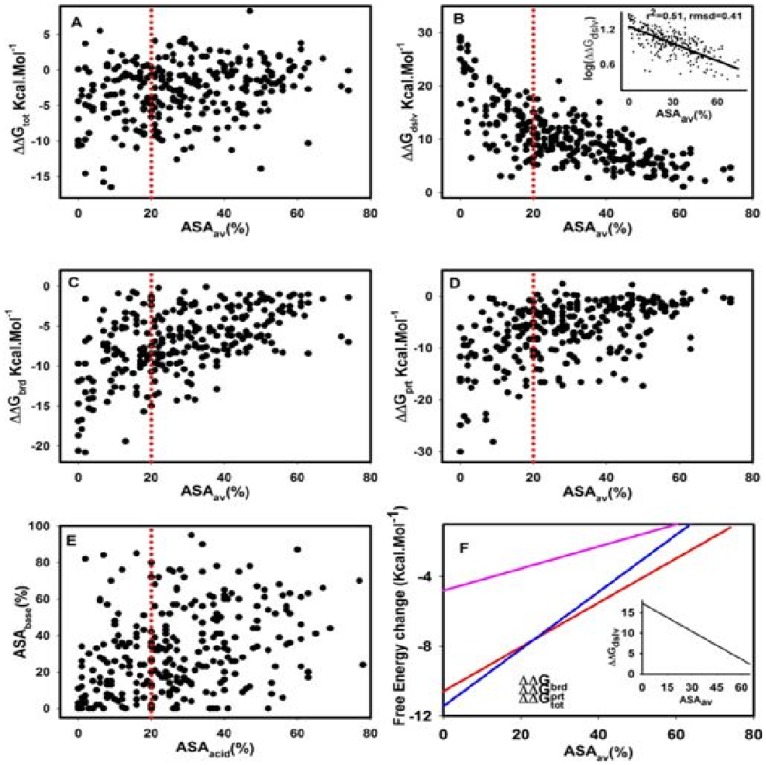
Analyses of correlation of ASA_av_ (%) (X-axis) with ΔΔG_tot_ (A), ΔΔG_dslv_(B), ΔΔG_brd_ (C) and ΔΔG_prt_ (D) for 275 salt-bridges from 20 halophilic proteins are presented. Correlation of accessibilities of individual salt-bridge partners is shown in E. Linear fit of logarithm of ΔΔG_dslv_ and ASAav (%) along with fitting parameters (correlation co-efficient and RMSD) are shown in the INSET of B. Plot F shows fitted lines for correlation of ΔΔGprt (blue), ΔΔGbrd (red) and ΔΔGtot (pink) with ASAav (%) along with that for ΔΔGdslv (in INSET of F).

To check relatedness among different energy terms with average accessible surface area (ASA_av_), correlation plots are presented in figure 4 (A through F) for all 275 candidate salt-bridges similar to earlier studies [29], [31]. **ΔΔG_dslv_**, which is energy change due to desolvation of salt-bridge partners due to folding, is linearly and negatively correlated with ASA_av_ (Figure 4 B).

The linear best fit of log of **ΔΔG_dslv_** against ASA_av_ could be expressed by the equation:
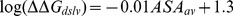
The fitted line has correlation coefficient 0.71 and RMSD 0.41 (INSET of Figure 4B). The linear correlation between ΔΔ**G_dslv_** and ASA_av_ with negative slope indicate transfer of salt-bridges into the core of protein involve greater desolvation penalty.

The dependence of observed data of **ΔΔG_brd_** (bridge term), **ΔΔG_prt_** (background term) and **ΔΔG_tot_** (net energy term) on ASA_av_ are shown in Figure 4: C, D and A respectively. Figure 4F shows such dependence by fitted lines only (without the observed data) for **ΔΔG_brd_**, **ΔΔG_prt_**, **ΔΔG_tot_** and ΔΔ**G_dslv_** (INSET of Figure 4F). From the figure, it is apparent that ΔΔ**G_dslv_** is negatively correlated (correlation coefficient r = 0.71) whereas both bridge and background terms are positively correlated (r = 0.51 and r = 0.48 respectively) with ASA_av_. Again, the curve for background term (blue line in Figure 4F) is seen to cross the bridge term (red line) around 22% of ASA_av_, indicating its stronger effect upon burial. However, such gain in energy upon burial of salt-bridges which is seen in both background and bridge terms were argued to be due to local environmental effect caused by linear gradient of dielectric constant and charge screening on protein surface (dielectric constant = 80) and in the interior (dielectric constant = 4) [29]. The fact of lower linear dependence (low correlation coefficient as above) of these two energy terms on ASA_av_; stronger background than bridge term upon burial (**ΔΔG** more negative of ASA_av_<20%) and more importantly large spread of individual salt-bridge energy of these terms against ASA_av_ (Figure 4 C and D) might indicate involvement of additional non-linear factors. As mentioned above, local microenvironment of a given salt-bridge could additionally and differentially be modulated by the presence of permanent dipoles (presence of peptide bond, helix, and polar groups) and non-specific side chain charges at neutral pH (not participating in salt-bridge) apart from the above uniform effect of dielectric constant mediated charge screening. In these aspects, the dependence of net energy term on ASA_av_ is very low (the correlation coefficient 0.07 pink lines Figure 4F) might be due to combined effect of its component terms.

### Stabilizing and destabilizing salt-bridges

Our database contains 275 salt-bridges obtained from 20 halophilic enzymes and proteins, making it suitable for understanding salt-bridge energetics. In table 3, salt-bridge energy classes (row: 1 to 5) are presented as buried or exposed (table 4) and stable or unstable (table 5) formats. In our dataset a total of 213 (78%) out of 275 salt-bridges possess **ΔΔG_tot_**<0, hence are stable. This observation, like Kumar and Nussinov (1999) and unlike Hendsch and Tidor (1994), shows majority of salt-bridges in our data set are stabilizing. The rest 62 (22%) salt-bridges are destabilizing type (Table 5, row 4). The instability in this population of salt-bridges are stemmed from the weaker contributions of background (which is weak by about 4.8 kcal mol^−1^) and bridge energy terms (weak by about 3 kcal mol; Table 3) than that of stable cases. Our observation is in line with earlier studies involving mesophilic proteins [29].

**Table 4:** Distribution of various salt-bridge classes under buried and exposed categories. Number outside indicate absolute count and percentage in parentheses.

Buried and exposed types in various salt bridge classes				
Salt bridge classes		Buried	Exposed	Total
**1**	**All**	94 (34.2%)	181 (65.8%)	275 (100%)
**2**	**Stable**	76 (35.7%)	137 (64.3%)	213(77.5%)
	**Unstable**	18 (29.0%)	44 (71.0%)	62(22.5%)
**3**	**Networked**	50 (40.0%)	75 (60.0%)	125(45.5%)
	**Isolated**	44 (29.3%)	106 (70.7%)	150(54.5%)
**4**	**H-bonded**	82 (36.3%)	144 (63.7%)	226(82.2%)
	**No H-bonds**	12 (24.5%)	37 (79.5%)	49(17.8%)

**Table 5:** Distribution of various salt-bridge classes under stable and unstable categories.

Stable and unstable types in various salt bridge classes				
Salt bridge class		Stable	Unstable	Total
**1**	**All**	213 (77.5%)	62 (22.5%)	275 (100%)
**2**	**Buried**	76 (80.9%)	18 (19.1%)	94(34.2%)
	**Exposed**	137 (75.7%)	44 (24.3%)	181(65.8%)
**3**	**Networked**	106 (84.8%)	19(15.2%)	125(45.5%)
	**Isolated**	107 (71.3%)	43 (28.7%)	150(54.5%)
**4**	**H-bonded**	186 (82.3%)	40 (17.7%)	226(82.2%)
	**No H-bonds**	27 (55.1%)	22 (44.9%)	49(17.8%)

Number outside indicate absolute count and percentage in parentheses.

How the buried and exposed salt-bridges contribute to the stability of halophilic proteins? Table 5 (row 3) shows out of 213 (78% of total 275) stable salt-bridges, 36% (76 of 213) are buried and remaining 64% are exposed. Similarly in unstable salt-bridges, 29% (18 of 62) are buried and remaining 71% are exposed. Interestingly, buried stabilizing population of salt-bridges is higher in our observations than observed in earlier study (present 36%; earlier 29%) by Kumar and Nussinov (1999). Nevertheless, overall distributions of buried and exposed salt-bridges under stable and unstable categories remain the same as earlier [43]. With respect to total buried (94 in Table 4), stable buried salt-bridges constitutes 81% (76 in 94) and remaining 19% are destabilizing type. At this point it is worth noting that only a little fraction of total destabilizing salt-bridges (18 of 62) are buried. In other words, of all buried salt-bridges (94 in table 4) very little fractions are destabilizing type (18 of 94). Statistical analyses of 275 salt-bridges by APBS methods (present studies) and 222 salt-bridges from mesophilic proteins by DELPHI software packages [29] show identical observation that majority of buried salt-bridges are stabilizing; are not in parallel with studies that involve selected salt-bridges and model systems [31], [44]. In this connection the contrast was argued to be due to the use of selected dataset and inclusion of ion-pairs with centroid distances greater than 4 Å [29].

Desolvation cost which was considered to be the sole factor for making buried salt-bridges always unstable [31], [44] may not be the only criteria for salt-bridge instability. In our study, a total of 44 out of 62 (Table 5) unstable salt-bridges, a greater proportion than burial ones (18 of 62), are seen to be exposed. How come destable salt-bridge populations are more under exposed than buried condition? Greater solvent mediated charge screening effect on salt-bridge charges (that affects both bridge and background terms) is apparent on the protein surface than in the solvent sequestered protein interior [29]. Further under halophilic situation, protein surface possess higher abundance of negative over positive charges, polar uncharged side chains (i.e. dipoles) than protein interior and thus unfavorable electrostatic interactions between biased proximity of these charges with surface salt-bridges (affect **ΔΔG_brd_** term); unfavorable charge-dipole interactions with surface salt-bridges (affect **ΔΔG_prt_** term) might cause reduction of favorable contributions of bridge and background terms. Additional factor that might contribute to buried salt-bridges being more stable is network salt-bridges (see below). In contrast to mesophilic situation [29], our dataset shows 50 of 76 total stable buried salt-bridges are network type (table 4).

### Buried and exposed salt-bridges

The location of salt-bridges in protein structure is determined by average accessibility [45], [46] of candidate salt-bridge partners, using a probe radius of 1.4 Å. An average ASA of ≤20% indicates the salt-bridge is in the core of protein otherwise exposed.

As far as contribution of buried salt-bridges is concerned, overall experimental observations showed that buried salt-bridges could be stabilizing, indifferent or mostly destabilizing [27], [32], [33], [47], [48]. Theoretical studies on buried salt-bridge also were shown to contribute little to protein stability. Thus there arises an apparent conflict about the contribution of buried salt-bridges: a point we consider below based on our and others observations [29], [43].

Computational analyses on salt-bridges in our dataset were classified into buried and exposed categories (table 4). 94 (34%) out of 275 salt-bridges are buried and rest 181 (66%) are exposed on proteins surface (Table 4). The unfavorable desolvation cost of these buried salt-bridges is indeed very high (i.e. 14.9 kcal mol^−1^) which is outweighed by joint effect of favorable bridge and background energy terms (Table 3 row 3). Interestingly, the favorable contribution of these two terms under buried condition exceed by about 4 kcal mol^−1^ than that under exposed condition making buried salt-bridges, on average, more stable. The overall stability of buried salt-bridges is seen to be −4.1±4.4 kcal mol^−1^ and that for exposed ones is −2.4±3.7 kcal mol^−1^ (Table 3 row 6). A similar observation was obtained in theoretical analyses involving 222 salt-bridges from 36 monomeric proteins [29].

The apparent conflict of buried salt-bridges with higher average net stability in our dataset that involve large population of candidate salt-bridges could get reasonable resolution if we consider the case on per protein basis. In studies with 38 protein structures, it was concluded that, on average, there exist 5 salt-bridges per 150 residues protein of which only 1 is under buried condition [43]. Similarly studies based on 36 proteins, it was shown that a total of 4 (222*150/9271) salt-bridges per 150 residues protein of which only 1 (55*150/9271) is under buried condition. Applying the above scale, our study with 20 halophilic proteins shows that a total of 8 (275*150/4975) salt-bridges per 150 residues protein with 3 (94*150/4975) under buried condition. It is thus apparent that buried salt-bridges on per protein basis are rare [43] in general. At this juncture it is worth noting that under halophilic condition, specific electrostatic make dominant contribution (78% and stable type) in that more number of salt-bridges and grater fraction of it is present in the protein interior than mesophilic ones. Such additional contribution of salt-bridges seems to be important under halophilic situation to compensate the deficit of low hydrophobic interactions [1].

However, unlike earlier studies [29] not all buried salt-bridges in our database are stable type. Out of 94, 76 (81%) buried salt-bridges are stable and that of 18 (19%) are unstable (table 4). In other words, on average, out of 3 buried salt-bridges in a 150 residues halophilic protein 1 is unstable and this figure is expected to be much narrower under mesophilic situation (see above). Thus it is apparent that finding stable buried salt-bridges depends on selection of i) type of protein and ii) candidate buried salt-bridge in that protein. Again, selection of buried salt-bridges is mostly limited due to the fact that it occurs in very low frequency in proteins in general (see above). Like earlier ones [29], [43], our study on large database shows that obtaining stable buried salt-bridge is purely context dependent.

How the buried salt-bridges overcome desolvation penalty and gain higher net stability? Although buried salt-bridges suffers from large desolvation penalty, entropic cost of localizing salt-bridges partners in the protein interior is minimized [49] at the same time. Hence buried salt-bridges are enthalpically favored. Again, in our database out of 94 buried salt-bridges, 50 form networked (Table 4). Under mesophilic condition, out of 66 buried salt-bridges only 6 are networked [29] indicating extensive network salt-bridges under halophilic situation. The fact that network salt-bridges contribute greater net (Table 3) stability (−5 kcal mol^−1^) and are mostly stable (85%; Table 5); halophilic situations seems to utilize the extensive networking to overcome desolvation penalty and thus to gain extra average net stability with buried salt-bridges. Apart from the above effects other important factor that might acts favorably, already noted above in the context of 275 salt-bridges, is the protein local microenvironments attributed with differential distribution of charges and dipoles in the vicinity of salt-bridges might modulate bridging and background terms to a varying degree. Overall the observations of highly stable buried salt-bridges indicate favorable contributions of these above factors.

### Isolated and networked salt-bridges in halophilic proteins

A salt-bridge between two oppositely charged residues is considered to be networked if at least one of these charged residues forms additional salt-bridge(s) with the other one(s). Otherwise, the salt-bridge is considered to be isolated. In our dataset, 125 of 275 (∼46%) salt-bridges are network type of which 40 triads, 12 tetrads, 1 pentad and 1 hexad are observed. The remaining 150 salt-bridges are isolated.

Salt-bridges mainly confer stability to tertiary structure of proteins [43]. However, their contribution to stability depends on their location, geometry and interactions of bridge partner's vicinity with other side chains in proteins [50]. While isolated salt-bridges provide marginal stabilization, network salt-bridges cause cooperative stabilization. In comparison to mesophilic proteins, in halophilic proteins higher level of network salt-bridges are observed. Halophiles like thermophiles are extremophiles. Our database shows 46% salt-bridges forming network and rest 54% are isolated type (Table 5). Such extensive network salt-bridges in halophilic proteins are not available with its mesophilic counterparts which form only 8% network salt-bridges [29] indicating extremophilic design in halophiles. Table 4 shows 50 (40%) of 125 network salt-bridges are buried which is greater than even isolated ones (29%). As far as stability is concerned, unlike mesophiles [29], 85% of network salt-bridges (106 out of 125, table 5) are stabilizing type and rest 15% is marginally unstable. In isolated case (Table 5), stable population constitutes 71% and that for unstable is 29%. Overall the net stability of network salt-bridges is about 4 times (Table 3) than that of isolated salt-bridges (network −4.97 kcal mol^−1^ and Isolated −1.28 kcal mol^−1^). Greater abundance and cooperative nature of network salt-bridges [51] seems to have greater significance under halophilic context. Sequence of halophilic proteins contains extra negative charges [1], [9] and in structures these residues are largely present on protein surface [37]. However, a sizable fraction of these excess charges are also present in the protein core. Network and isolated salt-bridge formation in protein core would satisfy these solvent sequestered charge residues. The fact that network salt-bridges contribute to higher stability and promote cooperative interactions among charges, halophilic design seems to utilize advantage of these interactions especially in their protein core such that obligatory presences of extra charges are satisfied. However, unlike earlier observation [43], the presence of destable population of network salt-bridges (15%) is not fully clear (table 5 row 4). A closer look on this population shows that lion's share of desolvation cost is balanced by bridge and background terms while making **ΔΔG_tot_** slightly positive. This phenomenon might indicate a trend towards maintenance of local flexibility a prerequisite for functionality, rather than stability and hence rigidity, is more critical.

### Hydrogen bonded and non-hydrogen bonded salt-bridges in halophilic protein

Salt-bridges in the dataset are also characterized based on their association with hydrogen atom. A hydrogen-bonded salt-bridge is identified by the presence of at least one pair of side-chain charged group atoms, with opposite partial charges, within a 3.5 A [29] distance. 226 out of 275 (82%, table 5) salt-bridges in our dataset contain at least one side-chain to side-chain hydrogen bond.

A total of 226 out of 275 (82%) salt-bridges in our dataset contain at least one hydrogen bond between bridging partner side-chains. The remaining 49 salt-bridges are devoid of such bond (Table 5). On average, the hydrogen bonded salt-bridges are more stabilizing than non-hydrogen bonded salt-bridges in that net energy gains (ΔΔG_tot_) for the former is −3 kcal mol^−1^ that for the later is −1.0 kcal mol^−1^ (table 3). The greater stability of H-bonded population of salt-bridges indicates excess electrostatic stabilization on salt-bridge self one. Similar net stabilizing effects are also expected due to salt-bridge proximity to dipoles or charges with modulation of component energy terms to some varying degrees. Thus, hydrogen bonded salt-bridges (as well as charges and dipole bonded ones) provides direct evidence for salt-bridges sensitivity to its local environment. However, hydrogen bonded salt-bridges have stronger bridge energy term than that of non-hydrogen bonded ones. The average ΔΔG_brd_ for the hydrogen bonded salt-bridges is −8 kcal mol^−1^, while that for the non-hydrogen bonded salt-bridges is −3 kcal mol^−1^ (Table 3). Like network salt-bridges, H-bonded salt-bridges are distributed both in the core and on the surface of proteins. A total of 82 out of 226 (36%) of the H-bonded salt-bridges are buried and 144 (64%) are exposed (table 4). As far as stability is concerned, 82% (186 out of 226) H-bonded salt-bridges are stabilizing and remaining 18% are destabilizing. Overall, formation of halophilic protein interior which is lack of bulky hydrophobic residues are contributed favorably both by network salt-bridge formation (see above) and hydrogen bonded salt-bridges.

## Conclusion

Our study with 275 salt-bridges from 20 halophilic proteins shows that about 80% of salt-bridges are conserved and contribute to halostability. In halophilic proteins, net salt-bridge energy is −3.0 kcal mol^−1^ and hence stable which is favored almost equally by bridge and background energy terms and disfavored by desolvation term. Both the former component terms are affected by factors: 1) dielectric constant of the medium and 2) local micro-environment of charges and dipoles in the vicinity of salt-bridges. While the former cause uniform modulation based on position of salt-bridge from the surface to the core; the later vary with context and composition of protein hence non-linear. Comparison between bridge and background terms for their energy contribution to 275 salt-bridges, the former contains 213 highly stable and 62 less stable energy populations, the later include 213 highly stable, 51 less stable and 11 unstable energy populations indicating the background term is more sensitive to protein local micro-environment. Specific polar nature of halophilic proteins over mesophilic ones seems to contribute to both background and bridge energy terms to overcome desolvation penalty and making net salt-bridge energy favorable. Halophiles recruits, on average, 8 salt-bridges per 150 residues protein which is almost double than that of mesophilic proteins. The fact that halophilic proteins have lower content of bulky hydrophobic residues and under saturated salt solution hydrophobic interactions but not the specific electrostatic ones are severely affected due to low water activity situation, additional salt-bridges compensates the deficit of hydrophobic force. These proteins possess higher proportion of buried salt-bridges than mesophiles and four-fifth of which are stable. Extensive networked salt-bridges have been another attribute of halophilic proteins of which 2/5 are found under buried condition. Halophilic protein interior is relatively more polar than their mesophilic counterpart, the cooperative networked salt-bridges in this protein core are crucial not only to overcome greater desolvation cost and thus making buried salt-bridges stable but also to satisfy isolated charges. Similar to networked salt-bridges hydrogen bonded salt-bridges also contribute to halostability. Under halophilic situation formation of protein core with relatively more polar residues, buried networked and hydrogen bonded salt-bridges play crucial role.

## Materials and Methods

### Dataset

We obtain 275 non-equivalent salt-bridges from 20 halophilic proteins following the definition of salt-bridges [46]. Every protein contains at least 50 residues. The three-dimensional structures of these proteins have been solved by X-ray crystallography, whose resolution is better than or equal to 1.4 Å and are available in the protein data bank (PDB) [44]. The PDB identity codes of these 20 halophilic proteins are **1DOI, 1ITK, 1MOG, 1MOJ, 1VDR, 2AZ3, 2IJQ, 2VWG, 2X98, 2ZUA, 3B73, 3CRJ, 3EEH, 3IFV, 3PUG, 3QTA, 3U1D, 4AF1, 4E19, 4JCO**.

### Salt-bridge extraction and categorization

A pair of oppositely charged residues (Asp or Glu with Arg, Lys or His) forms ion pairs in native protein structures. An ion pair is defined as a salt-bridge if they meet the following criteria: (i) The centroids of the side chain charged groups in oppositely charged residues lie within 4.0 Å of each other [43] and (ii) at least one pair of Asp or Glu side-chain carboxyl oxygen atoms and side-chain nitrogen atoms of Arg, Lys or His are within a 4.0 Å distance [25],[29]. The three-dimensional atomic coordinates of the charged atoms participating in ion pairs have been extracted from their respective PDB files. From the distances, ion pairs within 4 Å have selected for constructing our dataset of 275 salt-bridges. Of the observed salt-bridges, the percentage of individual basic and acidic residues involved in salt-bridge formation was calculated. The salt-bridge dataset is divided into several categories based on geometry i.e. hydrogen bonded or non-hydrogen bonded, location in the protein i.e. buried or solvent exposed and networking i.e. networked or isolated.

### Computation of continuum electrostatic energy contributions by salt-bridges

Energy contribution due to electrostatic interactions in proteins is computed using continuum electrostatics using APBS methodologies. PBE is a continuum description of electrostatics for proteins. This method models the protein as a low dielectric medium in which the charges of ionizable groups and the partial charges of permanent dipoles are assigned to the corresponding atoms according to the three-dimensional structures of the protein. The solvent is represented as a high dielectric medium and mobile ions are taken into account through the ionic strength. PB solver such as APBS method [36] and Delphi software package [24], [25], [28], [29], [31], [35] are popularly used for finding salt-bridge energies. Salt-bridge energy obtained by this *in silico* approach was found to be consistent with experimental observations [32], [41]. We have followed the former procedure and model as devised by Hendsch & Tidor, (1994) along with our in-house computational automations for obtaining values of different energy terms associated with salt-bridges. The electrostatic energy contribution (ΔΔ*G_tot_*) can be decomposed into three different energy terms: (i) ΔΔ*G_dslv_* is the energy difference caused by desolvation of charges. It is an unfavorable term. (ii) ΔΔ*G_prt_* is the energy difference due to background interactions of charge with permanent dipoles of the peptide backbone, of helices, or of non-ionizable polar side chains. (iii) ΔΔ*G_brd_* is the favorable bridge energy term that represents the electrostatic interaction between two charged residues side-chain groups in the folded state of the protein. First two terms are indirect and pH independent terms and the last one is direct and pH dependent term.

The total electrostatic energy contribution to the salt-bridge formation ΔΔ*G_tot_* is taken as sum of indirect and direct terms:
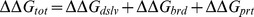
The electrostatic energy contribution to salt-bridge formation is calculated relative to a mutation of its salt-bridging side-chains to their hydrophobic isosteres. Hydrophobic isosteres are identical with the charged residue side-chains, with the exception that their partial atomic charges are set to zero. The energy minimization of initial structures is carried out with 100 steps of steepest descent followed by 500 steps of conjugate gradient using CHARMM force field in the GROMACS software. This procedure improves the accuracy of the continuum electrostatic calculations [45]. In each case, all hydrogen atoms are added, the protonation state of all charged residues are defined at pH 7.0, atomic radii and charges are assigned according to CHARMM force field using the program PDB2PQR [46]. Continuum electrostatic calculations are performed with the APBS [38]. The linearised PBE is solved on a 97×97×97 Å^3^ cubic grid box with finer grid spacing (0.5 Å per grid step) using iterative finite-difference methods [42], [43]. The solvent probe radius of 1.4 Å is used to define the molecular surface. The internal protein dielectric constant of 4.0 and the external solvent dielectric constant of 76 are used for each calculation. The ionic strength of 0.2M NaCl is used.

In each calculation, initially the molecule occupies 23% of the grid and the Debye-Huckel boundary conditions are applied. Results of this rough calculation are used as a boundary condition for a focused calculation in which the molecule occupies 92% of the grid. The results of the focused calculations are presented here. APBS outputs the energy values in units of κT, where κ is the Boltzmann constant and T is absolute temperature. These values are multiplied by a conversion factor of 0.592 to obtain the results in units of kilo calories per mole (Kcal mol^−1^) at room temperature (25°C).

### Database linking

PDB:**1DOI, 1ITK, 1MOG, 1MOJ, 1VDR, 2AZ3, 2IJQ, 2VWG, 2X98, 2ZUA, 3B73, 3CRJ, 3EEH, 3IFV, 3PUG, 3QTA, 3U1D, 4AF1, 4E19, 4JCO**.

## Supporting Information

Table S1 **Details of energetics of 275 salt-bridges from 20 extremely halophilic proteins.** The table also shows different salt-bridge classes (as in Table 3).
(XLSX)

## References

[1] Lanyi JK (1974) Salt-dependent properties of proteins from extremely halophilic bacteria. Bacteriol Rev 38: 272–290. 4607500 10.1128/br.38.3.272-290.1974 PMC413857

[2] Ginzburg M, Sachs L, Ginzburg BZ (1970) Ion metabolism in a Halobacterium. I. Influence of age of culture on intracellular concentrations. J Gen Physiol 55: 187–207. 5413077 10.1085/jgp.55.2.187 PMC2202994

[3] Eisenberg H (1995) Life in unusual environments: progress in understanding the structure and function of enzymes from extreme halophilic bacteria. Arch Biochem Biophys 318: 1–5. 7726549 10.1006/abbi.1995.1196

[4] Hecht K, Langer T, Wrba A, Jaenicke R (1990) Lactate dehydrogenase from the extreme halophilic archae bacterium Halobacterium marismortui. Biol Chem Hoppe-Seyler 371: 515–519. 2117936 10.1515/bchm3.1990.371.1.515

[5] Bandyopadhyay AK, Sonawat HM (2000) Salt Dependent Stability and Unfolding of [Fe2-S2] Ferredoxin of Halobacterium salinarum: Spectroscopic Investigations. Biophys J 79: 501–510. 10866976 10.1016/S0006-3495(00)76312-0 PMC1300954

[6] Rao JKM, Argos P (1981) Structural stability of halophilic proteins. Biochemistry 20: 6536–6543. 6796115 10.1021/bi00526a004

[7] Kennedy SP, Ng WV, Salzberg SL, Hood L, DasSarma S (2001) Understanding the adaptation of Halobacterium species NRC-1 to its extreme environment through computational analysis of its genome sequence. Genome Res 11: 1641–1650. 11591641 10.1101/gr.190201 PMC311145

[8] Bolhuis A, Kwan D, Thomas JR (2008) Halophilic adaptations of proteins. Protein adaptation in extremophiles. Nova Science Publishers Inc (USA) pp. 71–104.

[9] Paul S, Bag SK, Das S, Harvill ET, Dutta C (2008) Molecular signature of hypersaline adaptation: insights from genome and proteome composition of halophilic prokaryotes. Genome Biol 9: R70. 18397532 10.1186/gb-2008-9-4-r70 PMC2643941

[10] Tadeo X, Lopez-Mendez B, Trigueros T, Lain A, Castano D, et al (2009) Structural basis for the amino acid composition of proteins from halophilic archaea. PLoS Biol 7: e1000257. 20016684 10.1371/journal.pbio.1000257 PMC2780699

[11] Frolow F, Harel M, Sussman JL, Mevarech M, Shoham M (1996) Insights into protein adaptation to a saturated salt environment from the crystal structure of a halophilic 2Fe-2S ferredoxin. Nat Struct Biol 3: 452–458. 8612076 10.1038/nsb0596-452

[12] Mevarech M, Frolow F, Gloss LM (2000) Halophilic enzymes: proteins with a grain of salt. Biophys Chem 86: 155–164. 11026680 10.1016/s0301-4622(00)00126-5

[13] Pieper U, Kapadia G, Mevarech M, Herzberg O (1998) Structural features of halophilicity derived from the crystal structure of dihydrofolate reductase from the Dead Sea halophilic archaeon, Haloferax volcanii. Structure 6: 75–88. 9493269 10.1016/s0969-2126(98)00009-4

[14] Winter JA, Christofi P, Morroll S, Bunting KA (2009) The crystal structure of Haloferax volcanii proliferating cell nuclear antigen reveals unique surface charge characteristics due to halophilic adaptation. BMC Struct Biol 9: 55. 19698123 10.1186/1472-6807-9-55 PMC2737543

[15] Britton KL, Baker PJ, Fisher M, Ruzheinikov S, Gilmour DJ, et al (2006) Analysis of protein solvent interactions in glucose dehydrogenase from the extreme halophile Haloferax mediterranei. Proc Natl Acad Sci (USA) 103: 4846–4851. 16551747 10.1073/pnas.0508854103 PMC1458758

[16] Mevarech M, Eisenberg H, Neumann E (1977) Malate dehydrogenase isolated from extremely halophilic bacteria of the Dead Sea. 1. Purification and molecular characterization. Biochemistry 17: 3781–3785. 10.1021/bi00636a009 901751

[17] Bonete MJ, Pire C, Llorca FI, Camacho ML (1996) Glucose dehydrogenase from the halophilic archaeon Haloferax mediterranei: enzyme purification, characterization and N-terminal sequence. FEBS Lett 383: 227–229. 8925901 10.1016/0014-5793(96)00235-9

[18] Bandyopadhyay AK, Krishnamoorthy G, Sonawat HM (2001) Structural stabilization of [2Fe-2S] ferredoxin from Halobacterium salinarum. Biochemistry 405: 1284–1292. 10.1021/bi001614j 11170454

[19] Dym O, Mevarech M, Sussman JL (1995) Structural features that stabilize halophilic malate dehydrogenase from an archaebacterium. Science 267: 1344–1346. 17812611 10.1126/science.267.5202.1344

[20] Bandyopadhyay AK, Krishnamoorthy G, Padhy LC, Sonawat HM (2007) Kinetics of salt-dependent unfolding of [2Fe-2S] ferredoxin of Halobacterium salinarum. Extremophiles 4: 615–625. 10.1007/s00792-007-0075-0 17406782

[21] Elcock AH, McCammon JA (1998) Electrostatic contributions to the stability of halophilic proteins. J Mol Biol 280: 731–748. 9677300 10.1006/jmbi.1998.1904

[22] Dill KA (1990) Dominant forces in protein folding. Biochemistry 29: 7133–7155. 2207096 10.1021/bi00483a001

[23] Pace CN (1990) Conformational stability of globular proteins. Trends Biochem Sci 15: 14–17. 2107612 10.1016/0968-0004(90)90124-t

[24] Kumar S, Tsai CJ, Nussinov R (2000) Factors enhancing protein thermostability. Protein Eng 13: 179–191. 10775659 10.1093/protein/13.3.179

[25] Kumar S, Nussinov R (2001) How do thermophilic proteins deal with heat? Cell Mol Life Sci 58: 1216–1233. 11577980 10.1007/PL00000935 PMC11337401

[26] Horovitz A, Fersht AR (1992) Co-operative interactions during protein folding. J Mol Biol 224: 733–740. 1569552 10.1016/0022-2836(92)90557-z

[27] Marqusee S, Sauer RT (1994) Contribution of a hydrogen bond/salt-bridge network to the stability of secondary and tertiary structures in lambda repressor. Protein Sci 3: 2217–2225. 7756981 10.1002/pro.5560031207 PMC2142769

[28] Lounnas V, Wade RC (1997) Exceptionally stable salt-bridges in cytochrome P450cam have functional roles. Biochemistry 36: 5402–5417. 9154922 10.1021/bi9622940

[29] Kumar S, Nussinov R (1999) Salt-bridge stability in monomeric proteins. J Mol Biol 293: 1241–1255. 10547298 10.1006/jmbi.1999.3218

[30] Dao-pin S, Anderson DE, Baase WA, Dahlquist FW, Matthews BW (1991) Structural and thermodynamic consequences of burying a charged residue within the hydrophobic core of T4 lysozyme. Biochemistry 30: 11521–11529. 1747370 10.1021/bi00113a006

[31] Hendsch ZS, Tidor B (1994) Do salt-bridges stabilize proteins? A continuum electrostatic analysis. Protein Sci 3: 211–226. 8003958 10.1002/pro.5560030206 PMC2142793

[32] Waldburger CD, Schildbach JF, Sauer RT (1995) Are buried salt-bridges important for protein stability and conformational specificity? Nat Struct Biol 2: 122–128. 7749916 10.1038/nsb0295-122

[33] Barril X, Aleman C, Orozco M, Luque FJ (1998) Salt-bridge interactions: stability of ionic and neutral complexes in the gas phase, in solution and in proteins. Proteins: Struct Funct Genet 32: : 67 79. 10.1002/(sici)1097-0134(19980701)32:1<67::aid-prot8>3.0.co;2-b 9672043

[34] Dong F, Zhou HX (2002) Electrostatic contributions to T4 lysozyme stability: solvent-exposed charges versus semi-buried salt-bridges. Biophys J 83: 1341–1347. 12202359 10.1016/S0006-3495(02)73904-0 PMC1302232

[35] Li L, Li C, Sarkar S, Zhang J, Witham S, et al (2012) DelPhi: a comprehensive suite for DelPhi software and associated resources. BMC Biophys 4: 9. 10.1186/2046-1682-5-9 PMC3463482 22583952

[36] Guest WC, Cashman NR, Plotkin S (2010) Electrostatics in the stability and misfolding of the prion protein. Biochem Cell Biol 88: 371–381. 20453937 10.1139/o09-180

[37] Karan R, Capes MD, DasSarma S (2012) Function and biotechnology of extremophilic enzymes in low water activity. Aquatic Biosystems 8: 4. 22480329 10.1186/2046-9063-8-4 PMC3310334

[38] Baker NA, Sept D, Joseph S, Holst MJ, McCammon JA (2001) Electrostatics of nanosystems: application to microtubules and the ribosome. Proc Natl Acad Sci (U.S.A) 98: 10037–10041. 11517324 10.1073/pnas.181342398 PMC56910

[39] Baldwin RL, Rose GD (1999) Is protein folding hierarchic? II. Folding intermediates and transition states. Trends Biochem Sci 24: 77–84. 10098403 10.1016/s0968-0004(98)01345-0

[40] Tsai CJ, Lin SL, Wolfson HJ, Nussinov R (1997) Studies of protein-protein interfaces: a statistical analysis of the hydrophobic effect. Protein Sci 6: 53–64. 9007976 10.1002/pro.5560060106 PMC2143524

[41] Fleming PJ, Gong H, Rose GD (2006) Secondary structure determines protein topology. Protein Sci 15: 1829–1834. 16823044 10.1110/ps.062305106 PMC2242596

[42] Madigan D, Ryan PB, Schuemie M, Stang PE, Overhage JM, et al (2013) Evaluating the impact of database heterogeneity on observational study results. Am J Epidemiol 178: 645–651. 23648805 10.1093/aje/kwt010 PMC3736754

[43] Barlow DJ, Thornton JM (1983) Ion-pairs in proteins. J Mol Biol 168: 867–885. 6887253 10.1016/s0022-2836(83)80079-5

[44] Hendsch ZS, Sindelar CV, Tidor B (1998) Parameter dependence in continuum electrostatic calculations: a study using protein salt-bridges. J Phys Chem ser B 102: 4404–4410.

[45] Lee BK, Richards FM (1971) The interpretation of protein structures estimation of static accessibility. J Mol Biol 55: 379–400. 5551392 10.1016/0022-2836(71)90324-x

[46] Tsai CJ, Nussinov R (1997) Hydrophobic folding units derived from dissimilar monomer structures and their interactions. Protein Sci 6: 24–42. 9007974 10.1002/pro.5560060104 PMC2143523

[47] Lebbink JH, Consalvi V, Chiaraluce R, Berndt KD, Ladenstein R (2002) Structural and thermodynamic studies on a salt-bridge triad in the NADP-binding domain of glutamate dehydrogenase from Thermotoga maritima: cooperativity and electrostatic contribution to stability. Biochemistry 41: 15524–15535. 12501181 10.1021/bi020461s

[48] Marti DN, Bosshard HR (2003) Electrostatic interactions in leucine zippers: thermodynamic analysis of the contributions of Glu and His residues and the effect of mutating salt-bridges. J Mol Biol 330: 621–637. 12842476 10.1016/s0022-2836(03)00623-5

[49] Sun DP, Sauer U, Nicholson H, Matthews BW (1991) Contributions of engineered surface salt-bridges to the stability of T4 lysozyme determined by directed mutagenesis. Biochemistry 30: 7142–7153. 1854726 10.1021/bi00243a015

[50] Missimer JH, Steinmetz MO, Baron R, Winkler FK, Kammerer RA, et al (2007) Configurational entropy elucidates the role of salt-bridge networks in protein thermostability. Protein Sci 16: 1349–1359. 17586770 10.1110/ps.062542907 PMC2206687

[51] Albeck S, Unger R, Schreiber G (2000) Evaluation of direct and cooperative contributions towards the strength of buried hydrogen bonds and salt-bridges. J Mol Biol 298: 503–520. 10772866 10.1006/jmbi.2000.3656

[52] Lovell SC, Davis IW, Arendall WB 3rd, de Bakker PI, Word JM, et al (2003) Structure validation by Calpha geometry: phi, psi and Cbeta deviation. Proteins 50 3: 437–450. 12557186 10.1002/prot.10286

[53] Gupta PSS, Mondal S, Mondal B, Ul Islam RN, Banerjee S, et al (2014) SBION: A Program for Analyses of Salt-Bridges from Multiple Structure Files. Bioinformation 10 3: 164–166. 24748757 10.6026/97320630010164 PMC3974244

